# The effect of work-based mentoring on patient outcome in musculoskeletal physiotherapy: study protocol for a randomised controlled trial

**DOI:** 10.1186/1745-6215-15-409

**Published:** 2014-10-25

**Authors:** Aled L Williams, Ceri J. Phillips, Alan Watkins, Alison B. Rushton

**Affiliations:** Musculoskeletal Physiotherapy Service, Cardiff and Vale University Health Board, University Hospital of Wales, Heath Park, Cardiff, Wales CF14 4XW UK; Swansea Centre for Health Economics, College of Human and Health Sciences, Swansea University, Singleton Park, Swansea, Wales SA2 8PP UK; College of Medicine, Swansea University, Singleton Park, Swansea, Wales SA2 8PP UK; School of Sport, Exercise and Rehabilitation Sciences, College of Life and Environmental Sciences, University of Birmingham, Edgbaston, Birmingham, England B15 2TT UK

**Keywords:** Physiotherapy, Musculoskeletal, Clinical reasoning, Patient outcomes, Cost effectiveness, Education, Mentoring, Expertise

## Abstract

**Background:**

Despite persistent calls to measure the effectiveness of educational interventions on patient outcomes, few studies have been conducted. Within musculoskeletal physiotherapy, the effects of postgraduate clinical mentoring on physiotherapist performance have been assessed, but the impact of this mentoring on patient outcomes remains unknown. The objective of this trial is to assess the effectiveness of a work-based mentoring programme to facilitate physiotherapist clinical reasoning on patient outcomes in musculoskeletal physiotherapy.

**Methods/Design:**

A stepped wedge cluster randomised controlled trial (CRCT) has been designed to recruit a minimum of 12 senior physiotherapists who work in musculoskeletal outpatient departments of a large National Health Service (NHS) organization. Participating physiotherapists will be randomised by cluster to receive the intervention at three time periods. Patients will be blinded to whether their physiotherapist has received the intervention. The primary outcome measure will be the Patient-Specific Functional Scale; secondary outcome measures will include the EQ-5D, patient activation, patient satisfaction and physiotherapist performance. Sample size considerations used published methods describing stepped wedge designs, conventional values of 0.80 for statistical power and 0.05 for statistical significance, and pragmatic groupings of 12 participating physiotherapists in three clusters. Based on an intergroup difference of 1.0 on the PSFS with a standard deviation of 2.0, 10 patients are required to complete outcome measures per physiotherapist, at time period 1 (prior to intervention roll-out) and at each of time periods 2, 3 and 4, giving a sample size of 480 patients. To account for the potential loss to follow-up of 33%, 720 sets of patient outcomes will be collected.

All physiotherapist participants will receive 150 hours of mentored clinical practice as the intervention and usual in-service training as control. Consecutive, consenting patients attending treatment by the participating physiotherapists during data collection periods will complete outcome measures at baseline, discharge and 12 months post-baseline. The lead researcher will be blinded to the allocation of the physiotherapist when analyzing outcome data; statistical analysis will involve classical linear models incorporating both an intervention effect and a random intercept term to reflect systematic differences among clusters.

**Trial registration:**

Assigned 31 July 2012: ISRCTN79599220.

**Electronic supplementary material:**

The online version of this article (doi:10.1186/1745-6215-15-409) contains supplementary material, which is available to authorized users.

## Background

Outcomes research has been defined as the assessment of what does and does not work in the delivery of healthcare [1]. While a large volume of research has focussed on examining the effect of different treatment regimes on patient outcomes, it has been widely acknowledged that the clinician delivering treatment is an integral component of the intervention and that the interpersonal interactions between clinician and patient may have strong influences on outcomes [2–6]. This has led for calls to research the nature, type and extent of these interpersonal factors and to assess the degree to which changes in these factors can achieve better patient outcomes [2, 4]. It is also argued by researchers from within medical education that the education and development of clinicians should be evaluated in order to ascertain whether they achieve better health outcomes [1, 7–11]. Such evaluation would enable researchers to delineate how healthcare education contributes directly to the health of individuals and the public, improves the relevance and impact of medical education research, and enables patients and practitioners to make better-informed, cost-effective healthcare decisions [1, 10]. However, this type of research presents challenges, particularly in view of the multiple factors - physical, psychosocial, economic, environmental and cultural - that can influence patient outcomes [7, 12, 13]. Disappointingly, the call for research to investigate the effectiveness of educational interventions has been largely unheeded to date as there is a dearth of literature examining the impact of professional education on patient outcome [1, 7, 10, 14–17].

A key focus of postgraduate healthcare and medical education is that of expertise development [18–28]. The study of clinical expertise in healthcare has often relied on the assumption of experience being a critical factor [29–31]. As a result, much research has been performed with participating practitioners who have years of experience, or seniority, or who are expert by reputation [32–35]. This assumption has, however, been challenged in a study of expertise in musculoskeletal physiotherapy. Resnik and Jensen [36] used clinical outcomes to define expertise and were able to predict outcomes for patients after linear modelling. They did this by performing a retrospective analysis of health-related quality of life outcome data to calculate mean patient outcomes for each physiotherapist participating in an outcomes database and by using a generalized linear model to control for patient factors on outcomes such as patient age and severity of condition. By examining the differences between the actual and predicted outcome scores, an ‘expert group’ of therapists (the top 10% of therapists whose patients had the highest mean outcome scores) and an ‘average group’ of therapists (the 10% of therapists whose patients had 45^th^ to 55th percentile mean outcome scores) were identified. No difference in the years of clinical experience was found between these groups. The same authors have argued that the key behaviours of expertise can be identified, nurtured and taught [6, 37–40], and it therefore follows that if patient outcomes can be used to identify expert clinicians, the development of expertise within clinicians will contribute to improved patient outcomes. While key behaviours of physiotherapy expertise have been identified in qualitative studies, no empirical evidence has supported the development of such behaviours through education. However, interview data from physiotherapists who were deemed experts in the musculoskeletal field [36–38, 41] identified that experts attributed much of their development to working with mentors who facilitated their clinical reasoning processes: one of the identified behaviours of expert practice [36, 38, 39, 42, 43].

Clinical reasoning has been defined as a context-dependent way of thinking and decision making in professional practice that is used to guide practice actions [44], and research evaluating expertise in physiotherapy consistently identifies clinical reasoning as a critical component [37, 39, 40, 42, 44–46]. Expert clinicians have been shown to possess a broad scope of clinical reasoning strategies, and an ability to move between these different clinical reasoning strategies seamlessly [45, 47], using inductive and deductive thinking, collaborating throughout with their patients and informing - and being informed by - their practice knowledge and intervention strategies, ethical decisions and philosophies of practice [37, 38, 41, 42]. Experts’ clinical reasoning processes are able to move from the biomedical aspects of the patient’s presentations to the lived experience of that patient, and from diagnostic inquiry to instrumental and communicative management [43, 45]. In addition to studies of expert practice, the importance of clinical reasoning has been highlighted in work defining Master’s level clinical practice in healthcare in the United Kingdom. A population of tutors of all Master’s courses for healthcare professionals in the UK prioritised a high level of clinical reasoning skills as the most important behaviour for the construct of Master’s level clinical practice [48]. A high level of clinical reasoning skills as the most important behaviour was also identified in the subsequent study of the construct validity of Master’s level musculoskeletal physiotherapy [47]. Furthermore, a recent study into the research priorities for postgraduate theses in musculoskeletal physiotherapy internationally [49] − involving a sample of course tutors and expert clinicians nominated by Member Organizations of the International Federation of Orthopaedic Manipulative Physical Therapists (IFOMPT) - identified research questions investigating clinical reasoning processes and skills amongst the most important priorities. Authors in both the expertise and the clinical reasoning literature encourage researchers to perform studies of clinical reasoning in natural clinical settings [38, 43, 50–59]; therefore, research into the teaching and nurturing of the key behaviours of expertise - of which clinical reasoning is fundamental - should be set within the clinical context.

Education in the clinical context is highlighted from the small number of musculoskeletal studies that *have* employed educational interventions and assessed the clinical outcomes of patients treated by the participating clinicians. These studies can be divided into two groups. The first group of studies consist of cluster randomised controlled trials (CRCTs) evaluating the effect of implementation of a specific treatment approach (for example, new guideline approach to low back pain and psychosocial model of care) compared to usual care. The interventions all included education of the clinician but did not measure the efficacy of the educational programme *per se*; therefore, the evaluation of patient outcome was a measure of the combination of the treatment approach and the educational intervention. These cluster RCTs also explore the strengths and weaknesses of the educational interventions utilised. Interestingly, the findings of this group of studies are consistently disappointing in terms of impact on patient outcomes, and the authors frequently cite potential shortcomings in the education of the clinicians as a possible reason for the failure of the intervention to be more effective than usual care [60–64].

The second group of studies consists of two recent trials that specifically evaluated educational approaches using patient outcomes. Overmeer and colleagues [65, 66] investigated the impact of an 8-day university course for physiotherapists aimed at identifying and addressing psychosocial risk factors in patients with musculoskeletal pain and their effect on patient outcome. Their trial demonstrated that while the education programme did elicit statistically significant changes in clinicians’ attitudes, beliefs and knowledge, there was no impact on clinical patient outcomes, patient satisfaction, or perception of treatment [65]. Cleland and colleagues [67] investigated the impact on patient outcomes of an educational intervention to the treating physiotherapists. Clinical outcomes were measured for separate groups of patients treated by physiotherapists before and after a 2-day course on the management of neck pain. After the course, physiotherapists were randomised to intervention or control. In the intervention group, physiotherapists received ongoing education consisting of small group sessions and an educational outreach session where they received training in their clinical settings. The control group received no further education. While the changes in pain scores were not significantly different for patients treated by the two groups of physiotherapists, reductions in disability scores were significantly greater in patients treated by physiotherapists who received the additional ongoing training (mean difference of 4.2 points on the Neck Disability Index, *P* =0.019). The patients in the ongoing training group required a mean of 1.5 fewer visits during the post-training period, which was also statistically significant (*P* <0.001). While Cleland’s study [67] did not control for some variables (such as prognostic factors which could also have had an impact on patient outcome) and had the potential for contamination bias (physiotherapists from different groups worked in the same clinics), it was the first to demonstrate the effectiveness of an educational intervention on patient outcomes, and this merits further consideration. The length of training is cited as one potential reason for disappointing results in earlier studies [63], with time differences varying from 2 hours [61] to 8 days [65]. Cleland’s study [67] employed a 2-day course, two 1.5-hour meetings within the following 4 to 7 weeks, and a clinical outreach visit (1-hour co-treatment of a patient) followed by discussion, which is positioned between the two extremes for length of training. Two distinguishing features of Cleland *et al.*’s successful approach are that the education programme was ongoing and included an outreach visit with 1-hour co-treatment of an actual patient with neck pain in the physiotherapist’s own clinical practice setting. Overmeer and colleagues [65] propose that ongoing education within the clinical context may be the key element of an educational intervention, on the premise that to improve patient outcomes, changes in clinicians’ practice behaviour must result from the received educational intervention. The authors went on to suggest more explicitly that the educational strategy most likely to change practice behaviour is to educate the physiotherapist in the clinical environment and to provide direct clinical feedback on the clinician’s encounter with an actual patient.

This call for the delivery of education in the clinical context has also been argued elsewhere within the physiotherapy literature [68], in particular in Master’s level education where mentoring in the clinical environment is used nationally and internationally to develop clinical reasoning and other features of expert practice [69]. The rationale for this approach is outlined in the educational standards document of IFOMPT [69], a non-governmental International Manipulative Physical Therapy Federation representing international collaboration in musculoskeletal physiotherapy that is a recognised subgroup of the World Confederation for Physical Therapy, which in turn is a part of the World Health Organization (WHO). A minimum of 150 hours of mentored clinical practice is recommended for students, where the clinical mentor is a member of the Member Organization of IFOMPT. Furthermore, this clinical mentoring component of programmes has been explored in terms of its impact on physiotherapist performance and career and is consistently evaluated as an effective component of Master’s education on transforming practice through multiple qualitative studies [27, 28, 47–49, 70–73]. While the impact of this educational intervention has been explored on the physiotherapist, its impact on patient outcomes has not been investigated to date.

The objective of this trial is to assess the effectiveness and cost-effectiveness of a work-based mentoring programme for physiotherapists to facilitate clinical reasoning on patient outcomes in the field of musculoskeletal physiotherapy on patient clinical outcome.

## Methods/Design

**Figure 1:**
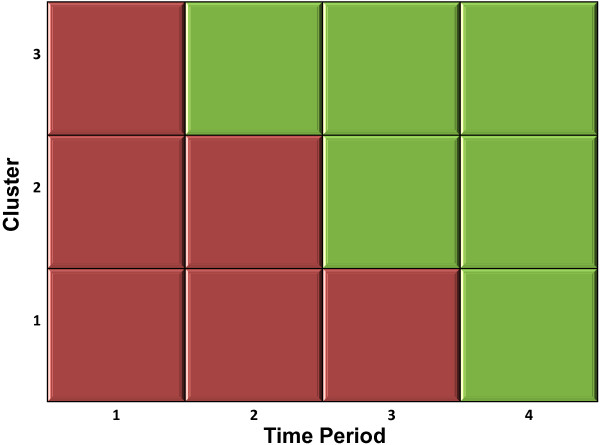
**The stepped-wedge design for this study.** Each cell represents a data collection point. Red cells represent control periods and green cells represent intervention periods.

A stepped-wedge design, a type of CRCT, has been designed in line with the SPIRIT 2013 statement [74]. A CRCT was selected because of the nature of the intervention and outcome. Educational interventions lend themselves to CRCT evaluation on the basis that for the training of clinicians to have an impact on their patients’ outcomes is appropriately a cluster-level intervention because education is frequently targeted at departments or practices [75–77]. In this study, the intervention will be applied at the cluster level to groups of physiotherapists (by department) – see Figure 1. The effect of the intervention on patient outcomes will also require measurement on clusters of patients treated by the physiotherapists before and after the intervention. Clustering assists in preventing contamination. An individual design would introduce the possibility of contamination by participating physiotherapists in different arms of the trial working together and discussing the intervention. By clustering physiotherapists by department and these clusters being the unit of randomisation, this risk of contamination is minimized [75, 77–79].

CRCTs can use parallel, crossover or stepped-wedge designs [80], and the stepped wedge design was selected for several reasons. First, there is not a belief of equipoise. That is, it is rational to believe that the intervention is likely to result in good rather than harm for both the participating physiotherapists, who will receive Master’s level mentoring, and the clusters of patients ‘exposed’ to management by the physiotherapists who have received training. This would make a parallel CRCT ethically less acceptable in that it would withhold the intervention from a larger proportion of physiotherapists and patients [81–83]. While a standard crossover CRCT would also satisfy this ethical concern, it would require the delivery of the intervention at the same time. This is logistically and practically difficult, as the intervention requires both specialist input and time input, making the stepped-wedge design preferable as an intervention that can be delivered in stages [81, 83, 84]. Second, the stepped-wedge design has been used to allow detection of underlying trends or control for time effects [81, 83]. This is of interest in the current study, as the multiple time points of data collection will allow for investigation of time effects to answer relevant questions. For example, does the effect dissipate over time, or does the intervention require time to consolidate and therefore have impact on patient outcomes? Third, the stepped-wedge design requires fewer clusters [84] than a parallel CRCT, and it maximises power as the intervention effect is estimated on both between-cluster and within-cluster comparisons [81].

The first step (time period 1) corresponds to a baseline measurement where none of the clusters receive the intervention. At each subsequent step, a cluster of participating physiotherapists (two departments of the musculoskeletal outpatient physiotherapy service) will cross over from control to receive the intervention.

### Participants

#### Eligibility criteria for participant clusters

All qualified physiotherapists working in the selected National Health Service (NHS) organization whose majority of time is practising in the musculoskeletal outpatient context will be eligible to participate. Exclusion criteria are physiotherapists who have already undertaken work-based placements as part of Master’s education, and rotational members of staff who will not be present for the duration of the study. Informed consent will be gained by approaching eligible physiotherapists and issuing detailed written information; those wishing to participate will give written consent, and they will be free to withdraw from the study at any time without giving reason.

#### Eligibility criteria for patient clusters

Consecutive consenting patients attending the outpatient musculoskeletal physiotherapy service for treatment by the participating physiotherapists during data collection periods will be eligible to participate. Exclusion criteria are patients under 18 years of age, and patients who are not English-literate due to the validity of some of the selected outcome measures being established only for the English language and for adults [85–92]. Patients will receive their normal care; written consent will be obtained for their outcome data to be used for the purposes of the study.

#### Settings and locations where the data will be collected

Patient outcome data will be collected at the first and last (discharge) appointment for physiotherapy treatment at six outpatient sites for musculoskeletal physiotherapy service delivery. The six sites will be organized into three pairs for training purposes, and these pairs will form the three clusters that will be the unit of randomisation as illustrated below. While the outcome at discharge will be the primary end point, patient outcome data at 12 months post-discharge will inform long-term impact on patient outcomes and assist in analysis of the cost-effectiveness of the intervention.

### Interventions

#### Intervention

The intervention is a 150-hour clinical mentorship programme, aimed at facilitating clinical reasoning, based on established practice in Master’s level courses in musculoskeletal physiotherapy, in line with the educational standards document of IFOMPT [69]. The focus on sound clinical reasoning for the educational intervention is supported by the educational standards [69]. Furthermore, a recent study into the research priorities for postgraduate theses in musculoskeletal physiotherapy internationally [49], identified prioritised research questions focused to clinical reasoning processes and skills. A focus on clinical reasoning is also supported by its highlighted importance in Master’s level clinical practice in healthcare in the United Kingdom [48], and specifically in Master’s level manipulative physiotherapy [47]. The educational intervention will be delivered to participating physiotherapists by mentors who are members of the UK Member Organization of IFOMPT, the Musculoskeletal Association of Chartered Physiotherapists (MACP), having undertaken extensive postgraduate study, having reached a recognised standard of excellence in musculoskeletal physiotherapy and having experience in delivering this form of mentorship at the postgraduate level. Once baseline data collection is completed in the first time period, the intervention will be rolled out, to one cluster per time period over the next three time periods. It will be delivered at the start of the time period to allow for consolidation and application of the programme before data collection occurs at the end of the time period. The intervention will take place in the usual clinical context of the participating physiotherapists. It will consist of the mentors observing the participating physiotherapists assessing and treating new and follow-up patients, and discussing and facilitating clinical reasoning processes immediately after the patient encounter.

#### Control

During the control steps of the study design, participants will receive their usual training allocation. Usual training for staff in the selected NHS organization involves monthly in-service training on current evidence applied to physiotherapy practice (4 hours per month), weekly technique sessions on the technical and practical skills of physiotherapy practice (30 minutes per week), as well as monthly mentoring sessions (1½ hours per month). While usual training does contain mentoring, the content, delivery and volume of this mentoring differs substantially from that of the intervention in that while patient interactions may be observed, they frequently take other forms, including practical skill teaching, tutorials, and retrospective reviews of patients using notes. Usual training allocates 1½ hours to mentoring, delivered once a month. In comparison, the intervention will allocate a much more intensive 150 hours of mentorship.

### Outcome measures

Clusters of patients will receive their physiotherapy treatment after the participating physiotherapists have received either intervention or control educational intervention. Patient outcome data will be collected at baseline (time period 1) and at the end of each of the subsequent three time periods (time periods 2 to 4) to allow for the consolidation and application of the training (which will have been delivered at the start of the time period).

Patient outcomes will be measured at the first visit, final discharge visit (primary end point), and at 12 months post-discharge, using four patient reported outcome measures. The primary outcome measure is health-related quality of life (HRQL) measured with a patient-specific tool, the Patient Specific Functional Scale (PSFS). Secondary outcome measures include a generic measure of HRQL (EQ-5D-5L), a measure of the patients’ ability to self-manage (the Patient Activation Measure (PAM)), and patient satisfaction (measured with the MedRisk instrument for Measuring Patient Satisfaction with Physical Therapy Care (MRPS)). In selecting appropriate outcome measures, three important factors were considered [93] - selection of dimensions to measure, psychometric properties, and practicality. The selection of dimensions to measure were influenced by the WHO’s International Classification of Functioning, Disability and Health (ICF) framework [94] for measuring health and disability at both individual and population levels [95–100].

The PSFS, a patient-specific HRQL measure, was selected as the primary outcome measure for its responsiveness [85, 101, 102] and psychometric properties (validity studies provide data on the minimal detectable change and the minimal clinically important difference across body regions [85, 87, 103–110], practicality [103] and dimensions selected for measurement, corresponding to the ICF dimensions of activity and participation being measured [111, 112]). The Euroqol (EQ-5D-5L) is the generic instrument selected to measure HRQL on the basis of its broad application to a wide range of health conditions and treatments and its provision of a simple descriptive profile and single index value for health status [113–118], which is appropriate for the variety of patients with different musculoskeletal conditions used in this study. The PAM will be used for the assessment of patient self-efficacy on the basis that one of the primary goals of rehabilitation is the enhancement of patient’s ability and confidence to manage their own health, which may not be fully captured by discharge functional or health related quality of life measures [37]. The PAM has undergone multiphase psychometric testing to confirm its validity and reliability and its ability to maintain precision across different demographic and health status groups [90, 91, 119–125]. Patient satisfaction will be measured, as one of the key features of development in expertise in clinical reasoning is collaborative reasoning [42, 43, 45] where patient centred care is seen as integral to practice. Moves towards patient centred care have seen the growth of interest in measurement of patient satisfaction as an important outcome measure in healthcare research [126, 127]. The MRPS will be used on the basis of its psychometric properties, its validation for use in an outpatient physiotherapy environment, its user-friendliness, and its satisfaction on the criteria identified by several authors exploring suitable patient satisfaction questionnaires [92, 127–133].

Physiotherapist performance will be assessed by an independent, blinded assessor who works outside of the NHS organization where the research is being conducted. This assessment of performance will be made using the criteria used by a UK academic institution experienced in the assessment of Master’s level postgraduate student performance in the musculoskeletal context. The independent assessor is educated to Master’s level and is MACP qualified, with several years experience of Master’s level mentoring, as well as experience in using the same criteria for assessment purposes.

### Sample size

Sample size calculations, using conventional values of 0.80 for statistical power and 0.05 for statistical significance, were performed following the approached outlined for stepped-wedge designs in Hussey and Hughes [84]. For a realistic sample of 12 participating physiotherapists, who will be organized into three clusters, and setting an intergroup difference of 1.0 on the PSFS with a standard deviation of 2.0 (based on previous patient data from the NHS organization and values of PSFS outcomes from published studies [87, 102, 134]), ten patients are required to complete the outcome measures per physiotherapist at each of the four time points. This will result in a total sample size of 480 sets of patient outcomes. In order to ensure a robust research protocol to ensure that adequate power is achieved, loss to follow-up rates were anticipated to be 33% at worst (based on data from the same organization from previous outcome collection), and the number of patients completing outcomes per physiotherapist per time point was revised to 15 to allow for this anticipated loss. The total required sample size was therefore established as 720 patients (that is, 12 physiotherapists collecting outcome measures from 15 patients at each of four time points).

### Randomisation and blinding

In stepped-wedge trials, the timing of intervention rollout is the unit of randomisation. The participating physiotherapists and clinical mentors implementing the mentoring interventions will be aware of which cluster is receiving the intervention [81, 82]. The clusters of participating physiotherapists (clustering by site) will be randomly allocated to the sequence of intervention (to receive the intervention in time period 2, 3 or 4), by computer programme [135]. This process will be performed by one of the clinical mentors, who will also allocate a unique identification code to each physiotherapist that will be included on all outcome measure questionnaires. The clinical mentor will keep a sealed copy of the key code linking codes to physiotherapists and will send a sealed copy of the key code to the academic institution responsible for trial governance.

The key code will only be opened once all outcome data have been collected and analyzed from each of the four time points. These processes will ensure allocation concealment and the blinding of the lead researcher to the sequence generation and intervention allocation. The independent assessor of physiotherapist performance will be blinded as to whether the physiotherapist has received the intervention during their assessment. Patients will also be blinded to whether or not their treating physiotherapist has received the intervention.

### Statistical methods

The statistical analysis of the stepped-wedge design has been an area of debate in the literature as analysis is said to be complex due to the need to account for repeated measures on the same individual and control for trends in outcome variables due to passage of time [81]. Indeed, in one systematic review of stepped-wedge CRCTs, the heterogeneity of analytical methods applied was seen as a weakness of some of the reviewed studies [83]. A standardised approach to analysis is recommended by the two systematic reviews. Such a standardized analysis of stepped-wedge trials has been clearly outlined in a published paper [84] and recommends processes for analysis of cluster-level means and individual level analysis in scenarios where cluster sizes are equal or unequal, and where variance is known or unknown. Processes for between-cluster and within-cluster analyses are outlined in order to avoid confounding the treatment effect with changes over time; these processes will form the basis for the statistical analysis for the study. If no temporal effects are found influencing the outcome, then a within-cluster analysis can be used to estimate the treatment effect. The proposed statistical analysis will involve classical linear models (so observations belong to control or intervention groups, differentiated by a single intervention effect), incorporating a random intercept term to reflect any systematic differences between clusters. Similar statistical methods have been used in other published stepped-wedge trials [81, 136, 137].

The use of aggregate differences in PSFS scores has been advocated and used in studies [134, 138], but has also been criticized [88, 139] on the basis that validity studies of the PSFS have been primarily related to changes in individual patients. These studies, however, also give important data on the minimal detectable change (MDC) and the minimal clinically important difference (MCID), which are both reported to be three points for each identified activity and two points for aggregate activities. This data is helpful, as analysis in this study will also look at the number or proportion of patients achieving significant changes in PSFS outcomes. This would require analysis of binary data, attributing 0/1 values to patients who do or do not achieve MDC/MCID. The convention for assessing the effect of clustering on binary variables is to assume normality and apply the usual linear models [140].

### Ethical approval

Ethical approval for the study was sought and obtained from the South East Wales Research Ethics Committee C on 20/04/2012 (ref: 12/WA/0078).

## Discussion

Stepped-wedge designs afford many advantages as outlined above. There are, however, limitations to this design, which are strongly emphasised by one group of authors in making their case for the superiority of standard parallel CRCTs [141]. The understanding of these disadvantages is not new, having been highlighted in two previous systematic reviews [81, 83] and reiterated in response to this criticism [142]. The limitations of the design are that the stepped-wedge design will take longer to conduct than a standard CRCT (due to the phased introduction of the intervention), the repeated measurements of the dependent variable increase the burden on participants and researchers, the potential risk of contamination or attrition in participants from a cluster due to receive the intervention at one of the later steps, and that an intervention is implemented in all clusters of the trial when it has not yet been proven to be effective. These points are important to address to clarify why the stepped wedge-design was selected over a standard parallel CRCT design for this trial.

The first point about the duration of the trial, and the associated point of attrition as a result of this, is well made. However, this trial is taking place in the real world of clinical practice, where normal service delivery and targets must still be met. While a standard parallel CRCT would reduce the time and potential attrition, it would be impractical for two reasons related to the intervention being delivered. First, the time factors involved in the intervention delivery would make the impact of introducing the intervention simultaneously across multiple clusters far greater on the capacity of the musculoskeletal physiotherapy service to deliver usual care across different clinics and venues. By rolling out the intervention across different time points, greater flexibility is afforded to organize clinic cover, allowing the participants to receive the intervention without compromise to the delivery of usual musculoskeletal service delivery commitments. Indeed, when negotiating the implementation of the RCT, the senior management within the organization made clear that the loss of the agreed clinical commitments required by a parallel CRCT would be unacceptable. Secondly, the delivery of the intervention is by mentors who have qualified at Master’s level, having received such mentoring as part of their postgraduate education, as well as delivering mentoring in this format to Master’s level students. Such a finite resource makes a standard parallel CRCT impractical, whereas the rolling out of the intervention in the stepped-wedge design utilises the available mentors in a way that makes this CRCT feasible.

Although the repeated measurements of a stepped-wedge design may create a burden on participants, similar measures are already collected regularly by the participants as standard clinical practice within the musculoskeletal physiotherapy service for its annual service evaluation process. The final point - that an intervention is implemented in all clusters of the trial when it has not yet been proven to be effective - is recognised as being valid in certain contexts. However, in the context of this trial, patients will receive their usual physiotherapy, and participating physiotherapists will receive an intervention for which there is already qualitative research supporting the impact of change in physiotherapist performance. Indeed, it could be argued that in a parallel CRCT, attrition from the control group could be greater than in the stepped-wedge CRCT design, as participating physiotherapists would in all likelihood concur that it is rational to believe that the intervention is likely to result in good rather than harm as discussed above.

In addition, the two systematic reviews raised further issues regarding the reporting and analysis of stepped-wedge CRCTs. A lack of fulfilment of the methodological requirements for a controlled trial, a lack of blinding of those assessing outcomes and heterogeneity of analytical methods applied were the key weaknesses identified. This led to the reviewers making the following recommendations for the reporting and analysis of stepped wedge CRCTs. First, authors should register their trial on the Controlled Clinical Trials Register and follow appropriate reporting guidelines; second, ways should be explored for enhancing internal validity through blinding of outcome assessors where possible and for the use of adequate sequence generation and allocation concealment; and third, standard methods of analysis should be used. The current study has been registered with Current Controlled Trials, and CONSORT and SPIRIT guidelines have been used in the protocol write-up. Sequence generation will be performed by one of the clinical mentors, randomly allocating the clusters of physiotherapists to the sequence of intervention by computer programme [135]. The allocated mentor will ensure that the sequence allocation is adequately concealed from the lead researcher, as all patient outcome data will be coded, the key code for which will be sealed and given to the academic institution responsible for the academic supervision of this research and not revealed until after the data collection and analysis period is completed. These measures for blinding, sequence generation and allocation concealment are implemented to reduce the risk of bias, as highlighted by the Cochrane risk of bias tool [143, 144]. With regard to statistical aspects, the approached outlined by Hussey and Hughes [75] for the design and analysis of stepped-wedge CRCTs has been adopted for sample size calculations and will be followed for analysis of the study data.

Through this RCT, the effectiveness of this mentoring programme for physiotherapists in the workplace to achieve better clinical outcomes for their patients will be evaluated. Specifically, this will allow the exploration of whether there are potential benefits to using such a training programme on a larger scale, as well as contributing to the body of literature on education and outcomes.

## Trial status

At the time of manuscript submission, the RCT had begun patient recruitment, which will not be complete until 2015.

## Authors’ information

AWi is clinical lead for Musculoskeletal Physiotherapy at Cardiff and Vale University Health Board. CP is Director of Research at the College of Human and Health Sciences and Professor of Health Economics at Swansea Centre for Health Economics, Swansea University. AWa is Senior Trial Statistician at the College of Medicine, Swansea University. AR is Academic Lead for Physiotherapy and Programme Leader of the MSc Exercise and Sport Medicine (Football) programme at the School of Sport, Exercise and Rehabilitations Sciences, University of Birmingham.

## Authors’ original submitted files for images

Below are the links to the authors’ original submitted files for images. Authors’ original file for figure 1 Authors’ original file for figure 2

## Abbreviations

CRCT: cluster randomised controlled trial
HRQL: health-related quality of life
ICF: International Classification of Functioning, Disability and Health
IFOMPT: International Federation of Orthopaedic Manipulative Physical Therapists
MRPS: MedRisk Instrument for Measuring Patient Satisfaction with Physical Therapy Care
MSK: Musculoskeletal
PAM: Patient Activation Measure
PSFS: Patient Specific Functional Scale
WHO: World Health Organization.
