# Supplementary figures and images for: The effect of work-based mentoring on patient outcome in musculoskeletal physiotherapy: study protocol for a randomised controlled trial
Source: Trials. 2014 Oct 25;15:409. doi: 10.1186/1745-6215-15-409 (PMC4223828; doi:10.1186/1745-6215-15-409)

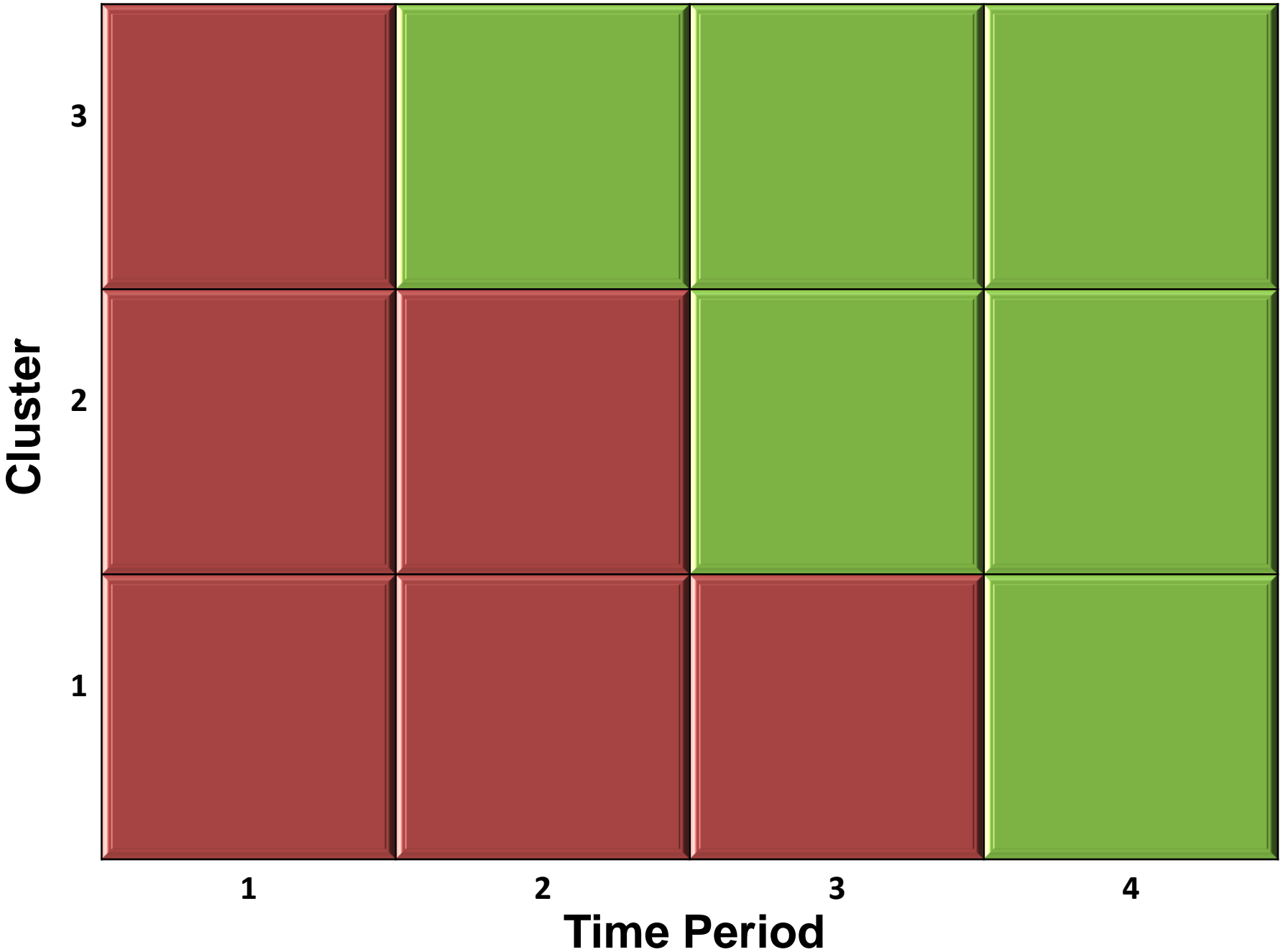

Supplement: Supplementary file 1 — Authors’ original file for figure 1 [file 13063_2014_2277_MOESM1_ESM.pdf]

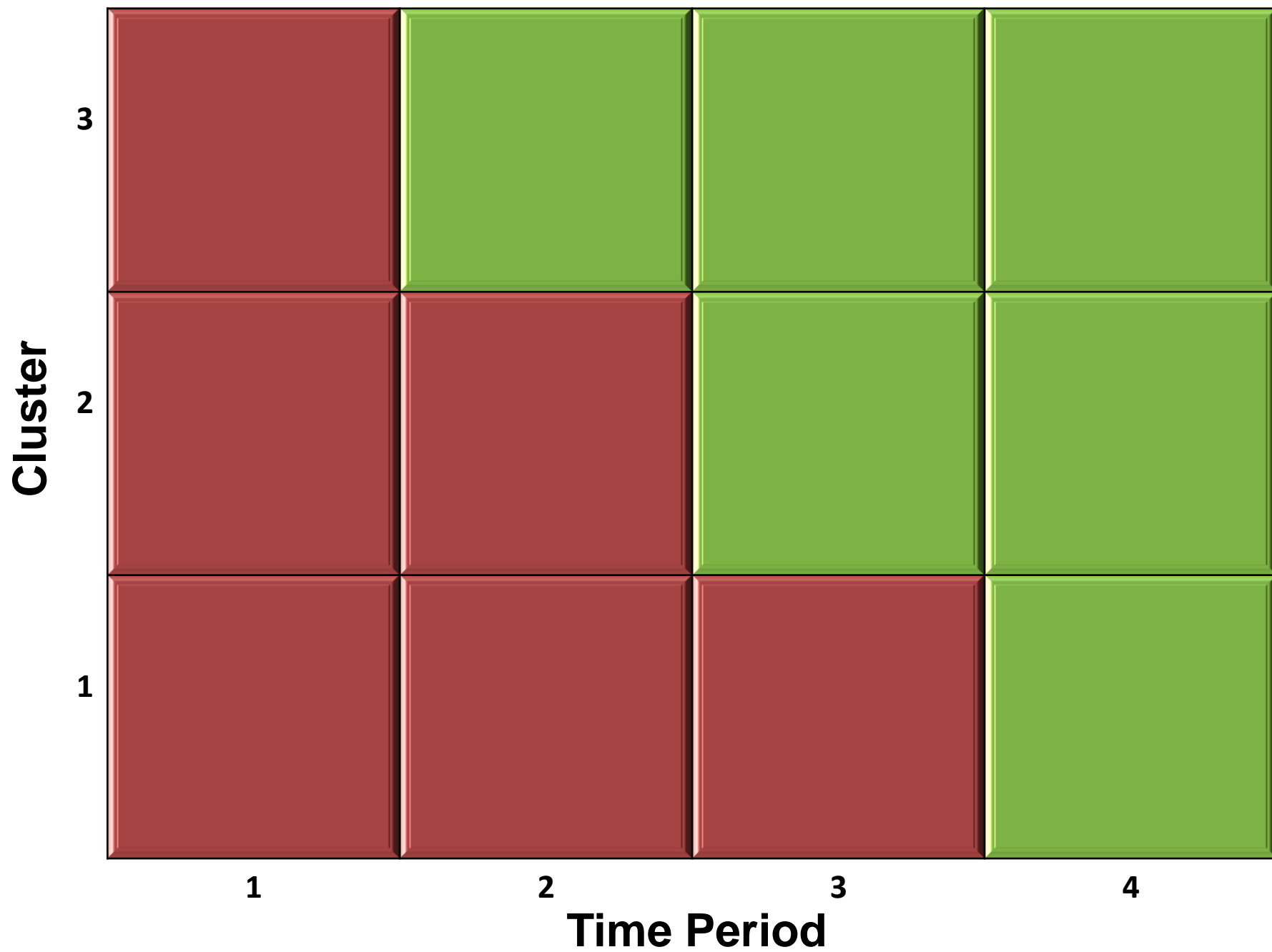

Supplement: Supplementary file 2 — Authors’ original file for figure 2 [file 13063_2014_2277_MOESM2_ESM.pdf]
